# Supplementary material for: Protein expression-based classification of gastric cancer by immunohistochemistry of tissue microarray
Source: PLoS One. 2020 Oct 23;15(10):e0238836. doi: 10.1371/journal.pone.0238836 (PMC7584200; doi:10.1371/journal.pone.0238836)
Supplement: Attachment — Submitted filename: Response to Reviewers.docx [file pone.0238836.s002.docx]

**Responses to Reviewers**

***Dear editors and reviewers:***

***Upon the submission of the revised manuscript entitled “A protein expression-based classification of gastric cancer by the Tissue Array Method immunohistochemistry”, we would like to thank you for your time and effort in evaluating this manuscript. Following the direction from the reviewers and the editor, we have now substantially revised this manuscript by addressing the main issues raised. We believe that the revision has significantly improved this study and the manuscript and hope it is now suitable for publication in Journal of PLOS ONE.***

(1) Title. Because the Title of this manuscript is not supported by results, you are advised to modify the Title.

Response: Thanks for pointing this out. We have now modified the title of our manuscript to more reflecting the findings as follow: “A protein expression-based classification of gastric cancer by the Tissue Array Method immunohistochemistry”

(2) Definition of mismatch repair deficiency (MMR-d). The authors defined MMR-d as “complete loss of expression in one of PMS2, MLH1, MSH2 or MSH6”; however, this definition is not a general definition in the global scientific community. You need to weaken their claim by using “MMR-d-like phenotype” or similar description.

Response: Thanks for your thoughtful suggestion. We have now replaced “MMR-d” with “dMMR-like” or “dMMR-like phenotype”

(3) Multi-variate analysis. You are advised to discuss the results of multivariate analysis as weak point in the abstract and discussion.

Response: Following your suggestion, we have now discussed the multivariate analysis results in the abstract and discussion sections (Page 2, lines 14-21; Page 17, lines 19-22; Page 18, lines 1-2).

(4) Other issues. You need to address other issues pointed out by Reviewer 1.

Response: Following the direction from the reviewer 1, we have now substantially revised this manuscript by addressing the main issues raised.

Reviewer #1: The authors developed a panel of immunohistochemical technology in a cohort of 259 GC patients and classified GC into 4 subtypes on the basis of expression of mismatch repair proteins (PMS2, MLH1, MSH2, and MSH6), E-cadherin and p21 protein. They observed that the subtypes presented distinct prognosis and corresponded to the molecular classifications previously reported. MMR-d subtype was associated with the best prognosis, and E-cadherin-a subtype was associated with the worst prognosis. Patients with p21-High and p21-Ligh subtypes had intermediate overall survival. Patients with the p21-High subtype most benefitted from adjuvant chemotherapy and those with the MMR-d subtype had the least benefit from adjuvant chemotherapy. There are several issues to be considered.

Major
1. The title of the manuscript is “Protein expression profiling identifies molecular subtypes of gastric cancer and predicts adjuvant chemotherapeutic benefit”. However, there are many cases who have not received adjuvant chemotherapy (N=123, 47.5%) and judging benefits of chemotherapeutic effects does not seem simple. So, it is better to delete those sentence from the title of the manuscript and more detailed statistical analyses are required to propose the authors’ hypothesis.
Response: Thanks for pointing this out. Following your suggestion, we have now eliminated the part about the chemotherapeutic benefit in the manuscript.

2. The main limitations of the present study are low and no significance observed in multivariate survival analyses in Table 2.
Response: Thanks for pointing this out. We have discussed the results of multivariate analysis as weak point in the Discussion as follow: “Our multivariate analysis has some limitations. In particular, some established prognostic factors of GC prognosis, notably Lauren type, Tumor differentiation, pN stage, and perineural invasion, did not reach significance may have been because these factors are co-linear with our classification in this study. Thus, the significance and robustness of the signature as a prognostic classification requires further confirmation with large prospective patient cohorts.” (Page 17, lines 19-22; Page 18, lines 1-2)

3. In Introduction, the authors described too much on gastric cancer incidences. Instead of this, it would better to add why the authors selected the antibodies used for this study.
Response: Following your suggestion, the entire Introduction section is carefully edited to reduce the description of gastric cancer incidences and add the reason for selecting the antibodies used. (Page 4, lines 2-6; Page 5, lines 1-6)

Minor
1. In page 5, TMA construction, “Two 2-mm-thick cores” can be replaced by “Two 2mm-sized cores”
Response: Corrected. Thanks.

2. The numbers of dMMR cases are more frequent than previously reported. Please explain the reason in Discussion.
Response: Following your suggestion, we have explained the reason in Discussion as follow: “In other papers, MSI was found in 5.6–30% of GC. Most of studies demonstrated that GCs with MSI were associated with advanced stage, elderly age, less lymph node involvement, intestinal type, and distal location. Considering that over 80 % of patients were diagnosed at an advanced stage of disease and nearly 70% of GCs were localized in the antrum, high proportion of dMMR cases may be acceptable to our study.” (Page 15, lines 20-22; Page 16, lines 1-4)

3. The authors described that “Digital images of TMA immunohistochemically stained slides were obtained via an Aperio Scanscope XT system (Leica Biosystems, Wetzlar, Germany)”. Then, why the authors did not use Aperio software to interpret them? It would be better to get the IHC data by digital image analyses instead of pathologists to reduce inter-observer variability.
Response: Thanks for pointing this out. This is the first time we use Aperio Scanscope XT system. Our technicians and pathologists currently lack training in the application of this Aperio Image Scope software. Although our pathologists have extensive experience in evaluation of IHC staining, the digital image analyses should be used to reduce inter-observer variability in our future studies.

4. In abstract, “corresponded to the molecular classifications previously reported” sentence seems inappropriate based on authors’ results.

 Response: Thanks for pointing this out. We have now eliminated this inappropriate sentence.
