# Supplementary material for: Protein expression-based classification of gastric cancer by immunohistochemistry of tissue microarray
Source: PLoS One. 2020 Oct 23;15(10):e0238836. doi: 10.1371/journal.pone.0238836 (PMC7584200; doi:10.1371/journal.pone.0238836)
Supplement: Attachment — Submitted filename: Response to Reviewers.docx [file pone.0238836.s003.docx]

**Responses to Reviewers**

Reviewer #1: The manuscript has been revised accordingly and improved. I have several minor comments.
1) In the title, please change "A protein expression-based classification of gastric cancer by the Tissue Array Method immunohistochemistry" to "Protein expression-based classification of gastric cancer by immunohistochemistry of tissue microarray".
Response：Thanks for pointing this out. We have now modified the title of our manuscript to more reflecting the findings as follow: “Protein expression-based classification of gastric cancer by immunohistochemistry of tissue microarray”

2) In Introduction, please add several efforts to replace molecular classifications of gastric cancer using archival tissue instead of fresh tissue such as nanostring (PMID: 29029513) or RNAseq of FFPE tissues (PMID: 31273278).
Response：Following your suggestion, we have now add several efforts in Introduction to replace molecular classifications of gastric cancer using archival tissue instead of fresh tissue (Page 5, lines 7-12).

3) In Discussion, please add limitations of the present study including "Many of the patients in this cohort have not received adjuvant chemotherapy, so our classifications could not predict the chemotherapeutic responses precisely.
Response：Following your suggestion, we have now add these limitations in Discussion sections (Page 18, lines 9-11).

Reviewer #2: This is a manuscript that provide molecular classification that is effective and inexpensive screening tool for improving prognostic models.
1. For patient with aberrant expression of mismatch repair protein (PMS2/ PMS2/MSH6/MSH2), did the author further explore if this is somatic or gremline?
Response：This is a very good question because experimentation to address this question would potentially provide additional evidence for this study. However, in order to publish these findings and thereby to stimulate more investigations from others in a timely fashion, we would like leave this rather time-consuming experiment for the future.

2. For patient with aberrant expression of mismatch repair protein (PMS2/ PMS2/MSH6/MSH2), it is helpful to know if they have received immunotherapy or not compared to other groups
Response：Thanks for pointing this out. According recent studies, immunotherapy has brought long-lasting tumor remission for gastric cancer, and mismatch repair deficiency (dMMR) as predictive biomarkers that guide the clinical application of immune checkpoint blockade therapies. As to our research, one of the criteria for recruiting patients is that the treatment plan is limited to chemotherapy. None of them had immunotherapy. As more and more gastric cancer patients receive immunotherapy in China, we would like focus on the relationship between dMMR subgroup and novel immunotherapy in our future studies.
